# Genetic and Phylogenetic Characterization of Influenza D Viruses From South Korean Cattle, 2022–2023

**DOI:** 10.1155/tbed/6780142

**Published:** 2026-08-05

**Authors:** Byunghyun An, Kyungmoon Lee, Hai Quynh Do, Junho Yoon, Songyi Kim, Eulhae Ga, Jong-Woo Lim, Minjoo Yeom, Dongjun An, Daesub Song

**Affiliations:** ^1^ Department of Virology, College of Veterinary Medicine and Research Institute for Veterinary Science, Seoul National University, Seoul 08826, Republic of Korea, snu.ac.kr; ^2^ Virus Disease Division, Animal and Plant Quarantine Agency, Gimcheon, Gyeongsangbuk-do, 39660, Republic of Korea, qia.go.kr

**Keywords:** bovine respiratory disease, D/Yama2019-lineage, influenza D virus, molecular epidemiology, phylogeographic analysis, South Korea, whole-genome sequencing

## Abstract

Influenza D virus (IDV) is an emerging orthomyxovirus with cattle as its principal reservoir, and D/Yama2019‐lineage viruses have become dominant in East Asia. Although IDV has been detected in Korean cattle, the genomic identity, phylogenetic placement, and regional evolutionary relationships of circulating Korean strains have not been defined. To address these gaps, nasal swabs were collected from 578 cattle with mild respiratory signs on 157 farms across eight provinces in South Korea during 2022–2023 and screened by RT‐qPCR targeting the PB1 gene. Positive samples underwent complete genome sequencing of all seven segments, followed by maximum‐likelihood and Bayesian phylogenetic analyses, discrete phylogeographic inference using a Bayesian stochastic search variable selection (BSSVS) model, and positive selection analyses. Six samples from three farms were IDV‐positive (sample‐level positivity: 1.04%; farm‐level positivity: 1.9%), all from 8‐to‐10‐month‐old calves. Phylogenetic analysis of all seven genomic segments placed all six Korean strains within the D/Yama2019 lineage with strong bootstrap support (99%–100%), forming a monophyletic cluster more closely related to Chinese than to Japanese D/Yama2019 reference strains. No phylogenetic evidence of reassortment was detected. Bayesian time‐scaled analysis estimated the most recent common ancestor of the Korean strains at ~2018.1–2020.1 across all seven segments. HEF‐based BSSVS analysis suggested a China‐to‐South Korea transition within the sampled dataset (posterior probability (PP) = 0.982; Bayes factor (BF) = 163.67), although this result should be interpreted in light of the small number of Korean sequences and the single‐segment basis of the phylogeographic analysis. Positive selection analyses revealed limited, method‐dependent signals without support from the fixed effects likelihood model, and Korean‐associated amino acid substitutions in PB1, P3, NS1, and NS2 were interpreted as lineage‐associated molecular signatures rather than evidence of adaptive evolution. These findings provide a whole‐genome baseline for IDV surveillance in South Korea and support continued longitudinal monitoring to clarify the persistence and transmission dynamics of D/Yama2019‐lineage viruses in the region.

## 1. Introduction

Influenza D virus (IDV) is an emerging orthomyxovirus first identified in swine and subsequently established as a distinct *deltainfluenzavirus* with cattle as its principal reservoir and amplification host [1, 2]. Molecular and serological surveillance has documented widespread IDV circulation across North America, Europe, and Asia [3–5]. Although IDV is not associated with high case fatality rates, its consistent detection in cattle with respiratory illness has raised concern about its contribution to bovine respiratory disease (BRD) in polymicrobial contexts, underscoring the importance of continued surveillance [6–8]. In an era of regional trade and dense production networks, surveillance of emerging respiratory viruses with transboundary potential is therefore essential.

The IDV genome comprises seven negative‐sense RNA segments, of which the hemagglutinin‐esterase‐fusion (HEF) gene encodes the sole surface glycoprotein shared with its closest relative, the influenza C virus [1, 2]. Unlike influenza A and B viruses, which encode separate hemagglutinin and neuraminidase proteins, the HEF protein integrates receptor‐binding, receptor‐destroying esterase, and membrane fusion activities within a single molecule, serving as the primary determinant of viral antigenicity and host cell tropism [9, 10]. Given its comparatively high sequence variability relative to the internal gene segments, HEF has been adopted as the principal genetic marker for IDV lineage classification.

The molecular epidemiology of IDV is complicated by ongoing genetic diversification and reassortment among co‐circulating lineages, including D/OK, D/660, D/Yama2016, D/Yama2019, and D/CA2019 [1, 2, 11–13]. In East Asia, D/Yama2019‐related viruses have emerged as the dominant clade, with recent studies in China and Japan documenting continued circulation, diversification, and interlineage reassortment [14–16]. Given this regional genetic context, the mere detection of IDV in a given country carries limited epidemiological value. Meaningful interpretation requires determining which lineage is circulating, how it relates to regional viral populations, and whether its presence reflects local endemicity, recent introduction, or transboundary dissemination.

The broad host range of IDV and its One Health implications further underscore the need for genomic surveillance. IDV can utilize both 9‐O‐acetylated N‐acetylneuraminic acid and N‐glycolylneuraminic acid as functional receptors, which may partly explain its expanded host tropism relative to more host‐restricted influenza viruses [9]. While IDV has not been established as a cause of human disease, experimental infection of human airway epithelial cells, serological evidence in cattle‐exposed populations, and molecular detection data collectively indicate that the cattle–human interface warrants continued attention [17–19].

In South Korea, IDV has previously been detected in cattle and swine [20], but the molecular epidemiology of circulating strains remains poorly defined. Critical gaps persist regarding the full genomic constellation of Korean IDV strains, their phylogenetic placement within the East Asian lineage landscape, their temporal relationship to viruses from neighboring countries, and the molecular signatures of adaptive evolution or selection. The relevant question is therefore no longer simply whether IDV is present in South Korea but which viral population is circulating and what its genomic features reveal about the history and trajectory of IDV in this region.

To address these questions, we characterized IDV from nasal swab samples of cattle with mild respiratory signs in South Korea using whole‐genome sequencing and molecular evolutionary analyses. We aimed to determine the genetic lineage of Korean IDV strains, reconstruct their phylogenetic relationships with global and East Asian reference viruses, estimate divergence times, infer geographic connectivity, and evaluate reassortment and selection signals across all genomic segments.

## 2. Materials and Methods

### 2.1. Sample Collection

**Figure 1:**
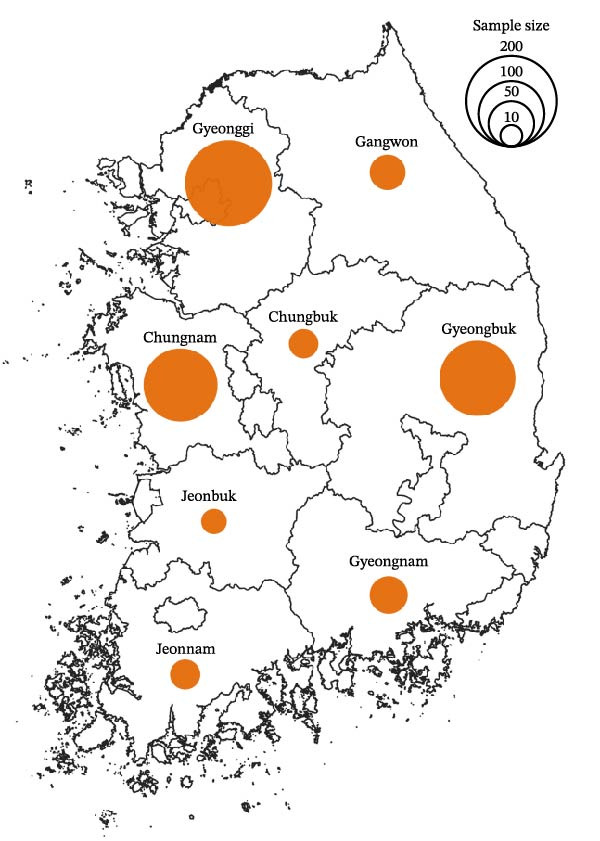
Geographic distribution of cattle sampled in South Korea during 2022–2023. The map shows the provincial distribution of cattle sampled from eight provinces. Orange circles indicate the number of animals sampled in each province, with circle size proportional to sample size. Samples were collected from Gyeonggi (182 animals from 44 farms), Gyeongbuk (139 animals from 25 farms), Chungnam (132 animals from 59 farms), Gyeongnam (35 animals from 11 farms), Gangwon (31 animals from 1 farm), Jeonnam (22 animals from 6 farms), Chungbuk (21 animals from 4 farms), and Jeonbuk (16 animals from 7 farms).

A total of 578 cattle aged 1–20 months exhibiting mild respiratory symptoms on 157 farms in South Korea during 2022–2023 were sampled, with two nasal swabs collected per animal. For each animal, the two nasal samples were placed together in a single tube containing Dulbecco’s modified Eagle medium (Welgene, Gyeongsan, Korea) supplemented with a 3% antibiotic–antimycotic solution (Gibco, USA) and were processed as one pooled sample for subsequent analysis. Samples were obtained from eight provinces (Figure 1): Gyeonggi (182 animals from 44 farms), Gyeongbuk (139 animals from 25 farms), Chungnam (132 animals from 59 farms), Gyeongnam (35 animals from 11 farms), Gangwon (31 animals from 1 farm), Jeonnam (22 animals from 6 farms), Chungbuk (21 animals from 4 farms), and Jeonbuk (16 animals from 7 farms).

### 2.2. RNA Extraction and RT‐qPCR Detection

Viral RNA was extracted from nasal swab samples using the RNeasy Mini Kit (Qiagen, Hilden, Germany) according to the manufacturer’s instructions. One‐step reverse transcription quantitative PCR (RT‐qPCR) was performed using the Luna Universal One‐Step RT‐qPCR Kit (New England Biolabs, Ipswich, MA, USA) with previously reported primers targeting the PB1 gene [21] (Supporting Information Table S1). Thermal cycling consisted of reverse transcription at 50°C for 30 min and initial denaturation at 95°C for 15 min, followed by 40 cycles of 95°C for 10 s and 60°C for 20 s.

### 2.3. Complete Genome Sequencing

IDV‐positive samples were selected for complete genome amplification of the seven genomic segments: polymerase basic 2 (PB2), polymerase basic 1 (PB1), polymerase 3 (P3), HEF, nucleoprotein (NP), matrix (M), and nonstructural (NS). Whole‐genome reverse transcription PCR (RT‐PCR) was performed with previously described primer sets [15, 22] (Supporting Information Table S1) using the PrimeScript II High Fidelity One‐Step RT‐PCR Kit (Takara, Shiga, Japan). RT‐PCR products were cloned prior to sequencing. Amplicons of the HEF, NP, P3, PB1, and M segments were cloned into the pTOP‐TA V2 vector, whereas those of the PB2 and NS segments were cloned into the pGEM‐T vector. Inserts in the pTOP‐TA V2 vector were sequenced using M13F and M13R primers, whereas those in the pGEM‐T vector were sequenced using T7 and SP6 primers. Of the six samples selected for complete genome sequencing, the PB2 segment from one sample and the NS segment from another sample were not successfully cloned and were therefore sequenced directly by Sanger sequencing.

### 2.4. BLAST Analysis and Reference Strain Selection

The obtained nucleotide sequences of each genomic segment were subjected to BLASTn analysis against the NCBI nucleotide database to identify closely related reference strains. BLAST hits were evaluated based on query coverage, percentage identity, and *E*‐value. The closest reference strain identified across the seven genomic segments was then used for subsequent pairwise nucleotide identity analysis.

### 2.5. Pairwise Nucleotide Identity Analysis

Pairwise nucleotide identities between the Korean IDV strains and the closest reference strain, D/bovine/CHN/JLSL/2021, were calculated for each genomic segment using MEGA version 7.1. Segment‐specific nucleotide alignments generated with MAFFT version 7.1 were imported into MEGA version 7.1, and pairwise distances were estimated using the *p*‐distance model [23–25]. Nucleotide identity was calculated as 100 × (1 − pairwise distance) and expressed as a percentage for each Korean strain and genomic segment.

### 2.6. Recombination Screening

Potential recombination events were screened for each genomic segment using Recombination Detection Programme 4 (RDP4) prior to downstream phylogenetic and selection analyses [26]. The analyses were performed using the RDP, GENECONV, BootScan, MaxChi, Chimaera, SiScan, and 3Seq methods implemented in RDP4. Recombination signals detected by at least four methods with Bonferroni‐corrected *p* values < 0.05 were considered statistically supported.

### 2.7. Maximum‐Likelihood Phylogenetic Analysis

For each genomic segment, reference IDV sequences were retrieved from the GenBank database (Supporting Information, Table S2) and subjected to multiple sequence alignment with the sequences generated in this study using MAFFT version 7.1 [23, 24]. The best‐fit nucleotide substitution model for each segment was selected in MEGA version 7.1 according to the Bayesian information criterion [25]. Maximum‐likelihood phylogenetic trees were reconstructed in MEGA version 7.1 using the selected model with 1000 bootstrap replicates [25]. Only bootstrap values of ≥80% are displayed at the nodes.

### 2.8. Bayesian Time‐Scaled Phylogenetic and Discrete Phylogeographic Analyses

To assess the temporal signal of each genomic segment, root‐to‐tip regression analysis was performed using TempEst version 1.5.3 [27]. Bayesian time‐scaled estimates from datasets with weak temporal signal were interpreted cautiously. Subsampling was not performed given the limited number and uneven distribution of dated IDV sequences across sampling years and geographic regions. Bayesian phylogenetic analyses were conducted in BEAST using segment‐specific nucleotide substitution and molecular clock models to account for differences in substitution patterns, evolutionary rates, and clock‐like behavior among genomic segments [28]. Candidate molecular clock models, including a strict clock, an uncorrelated relaxed clock with a lognormal distribution, and an uncorrelated relaxed clock with an exponential distribution, were compared for each segment using path sampling and stepping‐stone sampling [29, 30]. Bayes factors (BFs) calculated from marginal likelihood estimates were used to select the best‐supported molecular clock model for each dataset. A constant‐size coalescent tree prior was used for the primary analyses, and the selected molecular clock model was applied to each segment (Supporting Information, Table S3). Discrete phylogeographic inference was performed using a Bayesian stochastic search variable selection (BSSVS) model with four location states: South Korea, China, Japan, and other regions [31]. China and Japan were analyzed as separate states based on their phylogenetic proximity to the Korean D/Yama2019‐lineage HEF sequences, while the remaining regions were combined as other regions to reduce state complexity. The primary BSSVS analysis was conducted under a constant‐size coalescent prior. To evaluate whether the inferred transition support was robust to the choice of demographic prior, an additional BSSVS analysis was performed under a Bayesian skygrid coalescent prior as a sensitivity analysis. Support for each possible transition between location states was assessed using the posterior probability (PP) and BF of transition‐rate inclusion in the BSSVS model. PP indicates how often a given transition rate was included in the posterior samples, whereas BF measures how strongly the data supported that transition relative to the prior expectation. Following previous criteria, transitions were considered positively supported when BF > 3 and PP > 0.5, and strongly supported when BF > 20 and PP > 0.9.

The Markov chain Monte Carlo chain length was set to 1 × 10^8^, and the first 10% of samples were discarded as burn‐in. Convergence was assessed using Tracer, and effective sample size values >200 were considered indicative of adequate mixing. Maximum clade credibility (MCC) trees were summarized using TreeAnnotator. The time to the most recent common ancestor (tMRCA) and corresponding 95% highest posterior density (HPD) intervals were estimated from the posterior tree distributions, and tree topologies were visualized using FigTree version 1.4.

### 2.9. Positive Selection Analysis

Positive selection analyses were performed using the HyPhy package implemented on the Datamonkey web server, based on codon alignments and corresponding phylogenetic trees for each genomic segment [32, 33]. Selection patterns were assessed using the branch‐site unrestricted statistical test for episodic diversification (BUSTED) [34], adaptive branch‐site random effects likelihood (aBSREL) [35], mixed effects model of evolution (MEME) [36], and fixed effects likelihood (FEL) [37]. Results from these complementary methods were interpreted jointly. Statistical significance for selection analyses was defined as *p*  < 0.05.

### 2.10. Amino Acid Substitution Analysis

Deduced amino acid sequences of the Korean IDV strains were compared with those of representative reference IDV strains, with particular emphasis on Chinese and Japanese D/Yama2019‐lineage strains. Korean‐associated amino acid substitutions were identified across all genomic segments and summarized by segment.

## 3. Results

### 3.1. Detection and Genomic Characterization of IDV‐Positive Nasal Swab Samples

Among the 578 nasal swab samples collected from 157 cattle farms, six tested positive for IDV by RT‐qPCR, yielding an overall sample‐level positivity rate of 1.04% (6/578). At the farm level, IDV was detected in three farms, corresponding to a farm‐level positivity rate of 1.9% (3/157). Positive samples were detected in three provinces: Gyeonggi (*n* = 1), Chungnam (*n* = 2), and Gyeongbuk (*n* = 3). All positive animals were calves aged 8–10 months, comprising three 8‐month‐old calves from Gyeongbuk, one 9‐month‐old calf from Gyeonggi, and two 10‐month‐old calves from Chungnam (Table 1). Cycle threshold (*C*_t_) values ranged from 27.23 to 31.7. In Chungnam and Gyeongbuk, multiple positive samples were obtained from different animals within the same farm.

**Table 1:** Details of IDV‐positive cattle samples and corresponding GenBank accession numbers.

Strain name	Location	Age (months)	*C*_t_ value	GenBank accession no.
D/bovine/South Korea/SNU‐01/2022	Gyeongbuk	8	27.23	PX810064–PX810070
D/bovine/South Korea/SNU‐02/2022	Gyeongbuk	8	30.51	PX810071–PX810077
D/bovine/South Korea/SNU‐01/2023	Gyeongbuk	8	31.70	PX810078–PX810084
D/bovine/South Korea/SNU‐02/2023	Chungnam	10	28.04	PX810085–PX810091
D/bovine/South Korea/SNU‐03/2023	Chungnam	10	30.64	PX810092–PX810098
D/bovine/South Korea/SNU‐04/2023	Gyeonggi	9	31.29	PX810099–PX810105

Abbreviation: *C*_t_, cycle threshold.

Complete genome sequences comprising all seven genomic segments were successfully obtained for all six IDV‐positive strains. For five of the six strains, all segments were obtained via cloning‐based sequencing; however, the PB2 segment of one strain and the NS segment of another could not be successfully cloned and were therefore sequenced directly by Sanger sequencing. The complete genome sequences have been deposited in GenBank under accession numbers PX810064‐PX810105 (Table 1).

BLAST analysis identified D/bovine/CHN/JLSL/2021 as the closest reference strain to the six Korean IDV strains across all seven genomic segments. Pairwise nucleotide identity analysis showed that the Korean strains shared 98.54%–98.64% identity for PB2, 98.74%–98.83% for PB1, 99.14%–99.33% for P3, 98.56%–98.93% for HEF, 98.76%–99.07% for NP, 98.94%–99.21% for M, and 99.17%–99.58% for NS with D/bovine/CHN/JLSL/2021 (Table 2). Among the seven segments, PB2, PB1, and HEF showed relatively lower nucleotide identities, whereas P3 and NS showed consistently high identities across the Korean strains.

**Table 2:** Nucleotide identities between Korean IDV strains and the closest reference strain.

Korean IDV strain	Nucleotide identity with D/bovine/CHN/JLSL/2021 (%)						
	PB2	PB1	P3	HEF	NP	M	NS
D/bovine/South Korea/SNU‐01/2022	98.63	98.83	99.14	98.56	98.95	99.21	99.58
D/bovine/South Korea/SNU‐02/2022	98.63	98.83	99.33	98.66	98.76	99.21	99.58
D/bovine/South Korea/SNU‐01/2023	98.59	98.79	99.23	98.66	99.07	99.21	99.30
D/bovine/South Korea/SNU‐02/2023	98.54	98.74	99.14	98.87	99.01	98.94	99.30
D/bovine/South Korea/SNU‐03/2023	98.64	98.78	99.14	98.93	99.07	99.12	99.44
D/bovine/South Korea/SNU‐04/2023	98.54	98.74	99.23	98.92	99.07	99.12	99.17

### 3.2. Recombination Screening

Prior to phylogenetic and selection analyses, recombination screening was performed for each genomic segment using RDP4. No recombination signals were detected in any of the seven genomic segment alignments containing the Korean IDV sequences by the RDP, GENECONV, BootScan, MaxChi, Chimaera, SiScan, or 3Seq methods. Therefore, the original alignments were retained for subsequent maximum‐likelihood phylogenetic, Bayesian phylogenetic, phylogeographic, and positive selection analyses.

### 3.3. Phylogenetic Analysis of Korean IDV Strains

**Figure 2:**
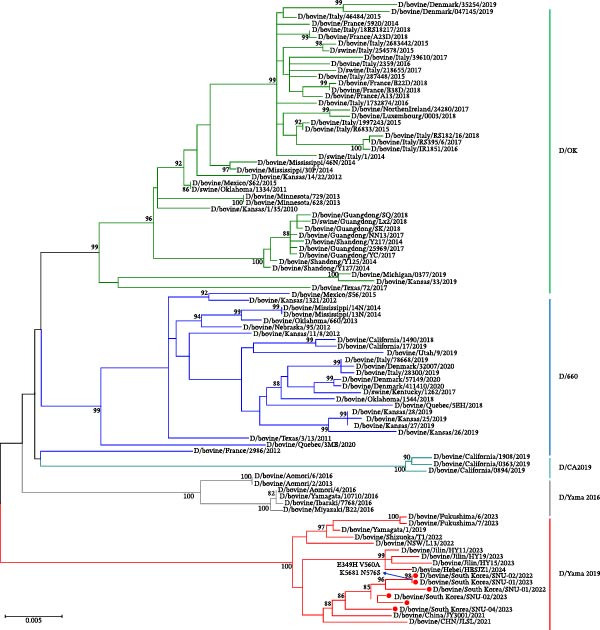
Maximum‐likelihood phylogenetic tree of the HEF gene segment of influenza D viruses. The six Korean IDV strains are indicated by red circles. Branch colors represent the major IDV lineages: D/Oklahoma (green), D/660 (blue), D/CA2019 (cyan), D/Yama2016 (gray), and D/Yama2019 (red). Bootstrap values ≥80% are shown at nodes. The scale bar indicates nucleotide substitutions per site.

Phylogenetic analysis of the HEF segment showed that all six Korean IDV strains clustered within the D/Yama2019 lineage with strong bootstrap support (100%) (Figure 2). This lineage also included IDV strains previously detected in cattle from China during 2021–2024 and from Japan during 2019–2023. Within this lineage, the Korean strains formed a distinct monophyletic cluster, separate from the Chinese and Japanese reference strains, but were phylogenetically closer to Chinese D/Yama2019 strains than to Japanese strains. In the HEF tree, two of the six Korean strains formed a minor subcluster characterized by four shared amino acid substitutions (E349H, V560A, K568I, and N576S). Consistent with the HEF results, phylogenetic analyses of the remaining six genomic segments (PB2, PB1, P3, NP, M, and NS) also placed all Korean strains within the D/Yama2019 lineage with strong bootstrap support (99%–100%) (Supporting Information Figure S1–S6). Across these segments, the Korean strains consistently formed a single cluster distinct from the Chinese and Japanese strains, while showing greater genetic relatedness to Chinese D/Yama2019 viruses. The concordant placement of the Korean strains across all seven segments, together with the absence of segment‐specific clustering with divergent IDV lineages, indicated no clear phylogenetic evidence of reassortment among the Korean strains.

### 3.4. Bayesian Time‐Scaled Phylogenetic and Phylogeographic Analyses

**Figure 3:**
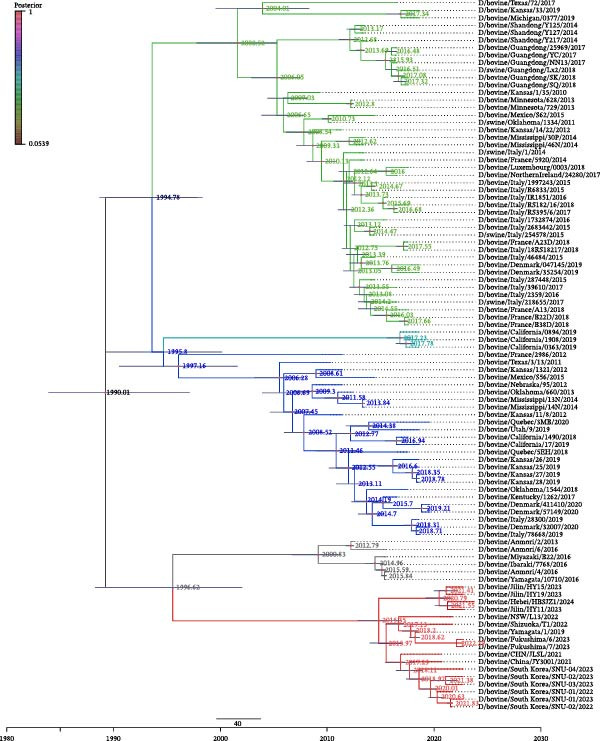
Bayesian maximum clade credibility tree of the HEF gene segment of influenza D viruses. The six Korean IDV strains clustered within the D/Yama2019 lineage, shown in red. Major IDV lineages are distinguished by branch color: D/Oklahoma (green), D/660 (blue), D/CA2019 (cyan), D/Yama2016 (gray), and D/Yama2019 (red). Node labels indicate estimated divergence times, and horizontal bars represent 95% highest posterior density intervals. Node colors indicate posterior probabilities. The time axis is shown in calendar years.

Bayesian time‐scaled phylogenetic analyses showed that the mean tMRCA estimates for the global IDV datasets differed by genomic segment but generally dated to the 1990s (Figure 3 and Table 3). TempEst root‐to‐tip regression indicated temporal structure in most genomic segments, although the signal was weaker for P3 and particularly limited for NP (Supporting Information Figure S7). The correlation coefficient and *R* ^2^ values were 0.71 and 0.26 for P3, and 0.27 and 0.0049 for NP, respectively. Therefore, time‐scaled estimates from these two segments, particularly NP, were interpreted cautiously and were not used as primary evidence for phylodynamic inference. Among all IDV strains, the earliest mean tMRCA was estimated for the HEF segment at 1989.4, whereas the most recent was estimated for PB1 at 1997.3. The mean nucleotide substitution rates for all IDV datasets were consistently on the order of 10^−3^ substitutions/site/year across the genomic segments. All Bayesian analyses achieved adequate MCMC convergence, with effective sample sizes exceeding 200 for all key parameters.

**Table 3:** Bayesian estimates of tMRCA and nucleotide substitution rates for each genomic segment of IDV.

Gene segment	No. of sequences	Mean tMRCA, year (95% HPD)		Mean substitution rates (substitutions/site/year, 95% HPD)
		All IDV	Korean IDV strains	
PB2	63	1992.5 (1982.4–2002.0)	2018.6 (2015.9–2019.3)	1.11 × 10^−3^ (8.57 × 10^−4^–1.36 × 10^−3^)
PB1	67	1997.3 (1990.6–2003.8)	2018.1 (2016.9–2019.5)	1.29 × 10^−3^ (1.01 × 10^−3^–1.61 × 10^−3^)
P3	74	1993.1 (1987.8–1998.2)	2018.3 (2017.2–2019.5)	1.08 × 10^−3^ (8.99 × 10^−4^–1.27 × 10^−3^)
HEF	95	1989.4 (1980.0–1997.8)	2018.1 (2016.8–2019.2)	1.19 × 10^−3^ (9.41 × 10^−4^–1.45 × 10^−3^)
NP	73	1996.6 (1985.6–2005.0)	2018.3 (2016.0–2018.6)	1.52 × 10^−3^ (1.11 × 10^−3^–1.95 × 10^−3^)
M	63	1997.2 (1984.0–2006.9)	2020.1 (2019.9–2022.3)	1.76 × 10^−3^ (1.10 × 10^−3^–2.49 × 10^−3^)
NS	63	1996.2 (1989.0–2002.4)	2019.9 (2017.5–2024.5)	1.12 × 10^−3^ (8.45 × 10^−4^–1.42 × 10^−3^)

Abbreviations: HPD, highest posterior density; tMRCA, time to the most recent common ancestor.

By contrast, the Korean IDV strains showed much more recent tMRCA estimates across all genomic segments, spanning 2018.1–2020.1. Most segments converged on a common ancestor around 2018, including PB1 and HEF (2018.1), PB2 (2018.6), and NS (2019.9), whereas M showed the most recent estimate (2020.1). P3 and NP also yielded recent tMRCA estimates of 2018.3, but these estimates should be interpreted cautiously because of their weaker temporal signal (Supporting Information Figure S8–S13). Overall, the broadly similar estimates across multiple segments suggest that the Korean IDV strains shared a relatively recent common ancestor.

To infer the phylogeographic history of the Korean HEF lineage, Bayesian discrete phylogeographic analysis with BSSVS was performed using the HEF segment and four location states: South Korea, China, Japan, and other regions (Table 4). Under the constant‐size coalescent prior, the China‐to‐South Korea transition was very strongly supported (PP = 0.982; BF = 163.67), whereas Japan‐to‐South Korea and Other‐to‐South Korea transitions were not supported. This inference was robust to demographic prior choice, as the Bayesian skygrid analysis yielded highly similar support for the China‐to‐South Korea route (PP = 0.981; BF = 154.89) and did not support alternative routes into South Korea. Together with the monophyletic placement of the Korean strains in the MCC tree, these results support a close relationship between the Korean HEF lineage and Chinese D/Yama2019‐lineage viruses based on the analyzed HEF sequences.

**Table 4:** Bayesian support for selected East Asian phylogeographic transition routes inferred from the HEF segment.

Route	Constant‐size		Skygrid		BSSVS support level
	PP	BF	PP	BF	
China → Korea	0.982	163.67	0.981	154.89	Strong support
Japan → China	0.542	3.55	0.539	3.51	Positive support
Korea → China	0.487	2.85	0.486	2.84	Not supported
Korea → Japan	0.43	2.26	0.428	2.24	Not supported
China → Japan	0.374	1.79	0.376	1.81	Not supported
Japan → Korea	0.319	1.41	0.322	1.42	Not supported
Other → Korea	0.198	0.741	0.199	0.745	Not supported

*Note:* “Other” denotes non‐East Asian sampling regions included as a fourth location state in the BSSVS analysis. BFs were calculated from the posterior and prior odds of inclusion for each transition rate. Transition routes were interpreted as showing positive support when BF > 3 and PP > 0.5, and strong support when BF > 20 and PP > 0.9.

Abbreviations: BF, Bayes factor; BSSVS, Bayesian stochastic search variable selection; PP, posterior probability.

### 3.5. Positive Selection Analysis

Positive selection analyses using BUSTED, aBSREL, MEME, and FEL identified limited evidence of episodic diversifying selection across the dataset (Table 5). BUSTED and MEME showed overlapping signals at PB2 codons 56, 79, 434, and 439; PB1 codon 744; HEF codons 233, 348, and 349; and NP codons 377, 465, and 511, whereas FEL did not support pervasive positive selection at any site. aBSREL detected branch‐specific episodic selection only on the branch leading to SNU‐01/2022 in the HEF tree. The substitution at PB2 439 was restricted to East Asian viruses, the substitution at PB1 744 was shared by most Korean strains and the Chinese JLSL/2021 isolate, and HEF 349 corresponded to the E349H substitution defining the minor Korean subcluster in the HEF tree. These patterns suggest episodic or lineage‐associated selection signals rather than broadly sustained adaptive evolution.

**Table 5:** Positive selection analyses across IDV genomic segments.

Positive selection model	Gene segment						
	PB2	PB1	P3	HEF	NP	M	NS
BUSTED	56, 79, 434, 439	744	—	233, 348, 349	377, 465, 511	—	—
MEME	56, 79, 434, 439	5, 744	106, 361	203, 233, 256, 264, 286, 348, 349, 456	377, 465, 511	—	—
FEL	—	—	—	—	—	—	—
aBSREL	—	—	—	SNU‐01/2022	—	—	—

*Note:* Values indicate codon positions detected by each selection analysis unless otherwise specified. A dash indicates no statistically significant signal. Statistical significance was defined as *p* < 0.05 for BUSTED, MEME, FEL, and aBSREL.

### 3.6. Korean‐Associated Amino Acid Substitutions

Amino acid comparison with Chinese and Japanese D/Yama2019‐lineage strains identified several Korean‐associated substitutions, including PB1 E99Q and A384T, P3 D395N and I483V, NS1 K80E, and NS2 N174H (Table 6). No Korean‐specific substitutions were observed in PB2, HEF, NP, or M. As these substitutions did not overlap with the selection‐supported sites, they likely represent molecular signatures of the Korean strains within the current East Asian D/Yama2019 dataset rather than direct evidence of adaptive evolution.

**Table 6:** Amino acid substitutions distinguishing Korean IDV strains from Chinese and Japanese D/Yama2019‐lineage strains.

Gene segment	Substitutions
PB1	E99Q, A384T
P3	D395N, I483V
NS1	K80E
NS2	N174H

## 4. Discussion

This study provides whole‐genome evidence that the Korean IDV strains belong to the D/Yama2019 lineage and form a coherent monophyletic cluster. These findings build on previous Korean evidence of IDV exposure by providing whole‐genome characterization of circulating Korean strains.

The low molecular detection rate is consistent with previous Korean and Japanese data but contrasts with recent Chinese surveillance. The first Korean survey detected IDV RNA in 1.4% of cattle, while seropositivity reached 54.7% [20]. Similarly, IDV RNA was detected in 2.1% of Japanese cattle [38]. By contrast, a recent Chinese survey reported RNA positivity of 11.1% and seropositivity of 91.4% in cattle [15]. The 1.04% detection rate in the present study, therefore, fits the pattern of low molecular positivity in Korea and Japan, while highlighting the discrepancy between active viral shedding and cumulative exposure. These findings suggest that cross‐sectional nasal swab testing captures only a limited window of IDV circulation.

IDV was detected only in 8‐to 10‐month‐old calves, an age range compatible with the post‐maternal‐antibody period described in cattle. In Mississippi beef cattle, maternal antibody levels declined substantially after 6 months of age, and transmission was observed among young, weaned, and commingled calves [39]. However, age‐stratified serological studies in Japan and Poland showed increasing IDV seropositivity with age, indicating cumulative exposure rather than restriction to a narrow calf age window [40, 41]. Thus, the present age distribution is compatible with detection after maternal antibody waning, but the small number of positive animals does not support defining 8–10 months as a specific risk period.

Previous studies identified D/Yama2019 as a distinct IDV lineage in Japan, and D/Yama2019‐like viruses were subsequently detected in Chinese cattle [12, 14–16]. Recent surveillance in China also reported the co‐circulation of D/Yama2019‐like viruses with D/660 lineage viruses [15]. In the present analysis, the Korean strains formed a Korean subcluster within the East Asian D/Yama2019 lineage and were genetically closer to Chinese D/Yama2019‐like strains than to the Japanese reference strains. The HEF‐based BSSVS result was consistent with this phylogenetic pattern, supporting regional linkage with Chinese D/Yama2019‐like viruses.

No evidence of reassortment was observed among the Korean strains. D/OK–D/660 reassortants have been reported in cattle from Quebec, Sweden, and Italy and in pigs from a mixed cattle–swine farm in France, indicating that reassortment is detectable in settings where divergent IDV lineages co‐circulate [8, 42–44]. By contrast, all seven genomic segments of the Korean strains grouped consistently within D/Yama2019, with no segment‐level phylogenetic discordance suggestive of reassortment. Recombination screening also did not identify statistically supported intrasegmental recombination signals.

The tMRCA estimates for the Korean strains were concentrated around 2018.1–2020.1, preceding their detection in 2022–2023. These estimates are more recent than previous tMRCA estimates for global IDV diversity and for the D/OK and D/660 lineages, and fall within the period of recent D/Yama2019 diversification reported in East Asia [12–16, 29]. The Korean strains therefore represent a recently diversified D/Yama2019‐related cluster, although the current data do not distinguish limited local circulation after introduction from repeated introductions of closely related viruses.

Selection analyses provided limited and method‐dependent evidence of episodic diversifying selection. Candidate signals were detected by BUSTED and MEME, whereas FEL did not support pervasive positive selection in any segment, and aBSREL detected branch‐specific episodic selection only on the branch leading to SNU‐01/2022 in the HEF tree. This pattern is consistent with previous IDV studies reporting selection signals mainly in HEF and less consistently in internal genes, while emphasizing that sequence‐based signals alone are insufficient to infer functional adaptation [29, 45]. Korean‐associated substitutions were identified in PB1 (E99Q, A384T), P3 (D395N, I483V), NS1 (K80E), and NS2 (N174H), but they showed little overlap with sites supported by selection analyses. Notably, the PB1 A384T substitution has previously been reported in D/OK and D/660 isolates from the USA and Canada [29], suggesting that this substitution is not exclusive to the Korean D/Yama2019 strains; given its proximity to W386, a residue involved in 5′‐terminal vRNA recognition, its functional significance merits further investigation. These substitutions are therefore best interpreted as lineage‐associated molecular signatures within the current East Asian D/Yama2019 dataset rather than evidence of host adaptation or antigenic change. Whether these substitutions affect viral phenotype, antigenicity, or host‐associated adaptation remains to be determined experimentally.

This study has several limitations. The cross‐sectional design and small number of complete genomes limit inference on transmission dynamics and rare reassortment events; however, the concordant whole‐genome placement of all Korean strains within D/Yama2019 provides robust support for the main phylogenetic conclusions. The absence of serological data and virus isolates prevents direct assessment of population‐level exposure and functional validation of the observed amino acid substitutions. These gaps are best addressed in future studies combining longitudinal serology, virus isolation, and experimental phenotypic characterization.

## 5. Conclusion

The Korean IDV strains identified in this study formed a recent D/Yama2019‐related cluster within the East Asian IDV population. Their consistent whole‐genome lineage assignment and lack of detectable reassortment provide a genomic baseline for Korean IDV surveillance. Further longitudinal surveillance integrating serology, virus isolation, and expanded whole‐genome sequencing is needed to clarify the persistence, transmission, and evolutionary dynamics of IDV in Korean cattle.

**Nomenclature**
IDV: Influenza D virus
RT‐qPCR: Reverse transcription quantitative PCR
PB1: Polymerase basic 1
BSSVS: Bayesian stochastic search variable selection
HEF: Hemagglutinin‐esterase‐fusion
P3: Polymerase 3
NS: Nonstructural protein.

## Author Contributions

**Byunghyun An:** conceptualization, methodology, investigation, writing – original draft, visualization, software, writing – review and editing. **Kyungmoon Lee:** methodology, visualization, software, data curation, writing – review and editing. **Hai Quynh Do:** writing – review and editing. **Junho Yoon:** writing – review and editing. **Songyi Kim:** writing – original draft, visualization, software, methodology. **Eulhae Ga:** writing – review and editing, formal analysis. **Jong-Woo Lim:** writing – review and editing. **Minjoo Yeom:** writing – review and editing, formal analysis, data curation. **Dongjun An:** investigation, software, writing – original draft. **Daesub Song:** writing – original draft, writing – review and editing, supervision, formal analysis, data curation, conceptualization.

## Funding

This research was supported by the Animal and Plant Quarantine Agency (APQA) and the National Institute of Wildlife Disease Control and Prevention, Gwangju, Republic of Korea, under the specialized Graduate School Support Project for Wildlife Disease Specialists. This work was also supported by the National Research Foundation (NRF) of Korea grant funded by the Korean government (Grant RS‐2024‐00432287).

## Ethics Statement

No ethical approval was required for this study. Nasal swab samples were provided by collaborating South Korean veterinarians as part of routine diagnostic surveillance of cattle with respiratory disease.

## Conflicts of Interest

The authors declare no conflicts of interest.

## Supporting Information

Additional supporting information can be found online in the Supporting Information section.

## Supporting information


**Supporting Information** Table S1 lists the primers and probes used in this study. Table S2 provides the GenBank accession numbers, strain names, and collection years of the reference influenza D virus sequences included in the phylogenetic analyses. Table S3 summarizes the molecular clock and nucleotide substitution models used for each genomic segment. Figures S1–S6 present maximum‐likelihood phylogenetic trees for the PB2, PB1, P3, NP, M, and NS segments; Figure S7 presents the root‐to‐tip regression analyses; and Figures S8–S13 show the corresponding Bayesian maximum clade credibility trees.

## Data Availability

The data presented in this study are available from the corresponding author upon reasonable request. GenBank accession numbers PX810064–PX810105 encompass the complete genome sequences of D/bovine/South Korea/SNU‐01/2022 through D/bovine/South Korea/SNU‐04/2023.

## References

[1] Hause B. M. , Ducatez M. , and Collin E. A. , et al.Isolation of a Novel Swine Influenza Virus From Oklahoma in 2011 Which Is Distantly Related to Human Influenza C Viruses, PLoS Pathogens. (2013) 9, no. 2, 10.1371/journal.ppat.1003176, e1003176. 23408893 PMC3567177

[2] Hause B. M. , Collin E. A. , and Liu R. , et al.Characterization of a Novel Influenza Virus in Cattle and Swine: Proposal for a New Genus in the *Orthomyxoviridae* Family, mBio. (2014) 5, no. 2, e00031–e00014, 10.1128/mBio.00031-14. 24595369 PMC3958797

[3] Yu J. , Li F. , and Wang D. , The First Decade of Research Advances in Influenza D Virus, Journal of General Virology. (2021) 102, no. 1, 10.1099/jgv.0.001529, 001529. PMC8279278 33211641

[4] Ruiz M. , Puig A. , Bassols M. , Fraile L. , and Armengol R. , Influenza D Virus: A Review and Update of Its Role in Bovine Respiratory Syndrome, Viruses. (2022) 14, no. 12, 10.3390/v14122717, 2717. 36560721 PMC9785601

[5] Kwasnik M. , Rola J. , and Rozek W. , Influenza D in Domestic and Wild Animals, Viruses. (2023) 15, no. 12, 10.3390/v15122433, 2433. 38140674 PMC10748149

[6] Nissly R. H. , Zaman N. , and Ibrahim P. A. S. , et al.Influenza C and D Viral Load in Cattle Correlates With Bovine Respiratory Disease (BRD): Emerging Role of Orthomyxoviruses in the Pathogenesis of BRD, Virology. (2020) 551, 10–15, 10.1016/j.virol.2020.08.014. 33010670 PMC7519714

[7] Lion A. , Secula A. , and Rançon C. , et al.Enhanced Pathogenesis Caused by Influenza D Virus and *Mycoplasma bovis* Coinfection in Calves: A Disease Severity Linked With Overexpression of IFN-γ as a Key Player of the Enhanced Innate Immune Response in Lungs, Microbiology Spectrum. (2021) 9, no. 3, 10.1128/spectrum.01690-21, e0169021. 34937196 PMC8694133

[8] Saegerman C. , Gaudino M. , and Savard C. , et al.Influenza D Virus in Respiratory Disease in Canadian, Province of Québec, Cattle: Relative Importance and Evidence of New Reassortment between Different Clades, Transboundary and Emerging Diseases. (2022) 69, no. 3, 1227–1245, 10.1111/tbed.14085. 33764631

[9] Uprety T. , Sreenivasan C. C. , and Yu J. , et al.Differential Tissue Tropism and Transmission Efficiency of Two Dominant Influenza D Clades With Overlapping but Distinct Receptor Binding Fine Specificities in Ferrets, PLoS Pathogens. (2025) 21, no. 9, 10.1371/journal.ppat.1013493, e1013493. 40953060 PMC12435653

[10] Song H. , Qi J. , and Khedri Z. , et al.An Open Receptor-Binding Cavity of Hemagglutinin-Esterase-Fusion Glycoprotein From Newly-Identified Influenza D Virus: Basis for Its Broad Cell Tropism, PLoS Pathogens. (2016) 12, no. 1, 10.1371/journal.ppat.1005411, e1005411. 26816272 PMC4729479

[11] Collin E. A. , Sheng Z. , Lang Y. , Ma W. , Hause B. M. , and Li F. , Cocirculation of Two Distinct Genetic and Antigenic Lineages of Proposed Influenza D Virus in Cattle, Journal of Virology. (2015) 89, no. 2, 1036–1042, 10.1128/JVI.02718-14. 25355894 PMC4300623

[12] Murakami S. , Sato R. , Ishida H. , Katayama M. , Takenaka-Uema A. , and Horimoto T. , Influenza D Virus of New Phylogenetic Lineage, Japan, Emerging Infectious Diseases. (2020) 26, no. 1, 168–171, 10.3201/eid2601.191092. 31855532 PMC6924913

[13] Gaudino M. , Chiapponi C. , and Moreno A. , et al.Evolutionary and Temporal Dynamics of Emerging Influenza D Virus in Europe (2009-22), Virus Evolution. (2022) 8, no. 2, 10.1093/ve/veac081, veac081. 36533151 PMC9752663

[14] Yu J. , Li T. , and Wen Z. , et al.Identification of D/Yama2019 Lineage-Like Influenza D Virus in Chinese Cattle, Frontiers in Veterinary Science. (2022) 9, 10.3389/fvets.2022.939456, 939456. 35909676 PMC9330358

[15] Yu J. , Wen Z. , and Hu W. , et al.Influenza D Virus Infection in China, 2022–2023, Emerging Microbes and Infections. (2024) 13, no. 1, 10.1080/22221751.2024.2343907, 2343907. 38738553 PMC11097708

[16] Li H. , Yan W. , and Liu X. , et al.Emergence and Phylodynamics of Influenza D Virus in Northeast China Reveal Sporadic Detection and Predominance of the D/Yamagata/2019 Lineage in Cattle, Viruses. (2026) 18, no. 1, 10.3390/v18010093, 93. 41600857 PMC12846649

[17] Holwerda M. , Kelly J. , and Laloli L. , et al.Determining the Replication Kinetics and Cellular Tropism of Influenza D Virus on Primary Well-Differentiated Human Airway Epithelial Cells, Viruses. (2019) 11, no. 4, 10.3390/v11040377, 377. 31022887 PMC6521319

[18] Leibler J. H. , Abdelgadir A. , and Seidel J. , et al.Influenza D Virus Exposure Among US Cattle Workers: A Call for Surveillance, Zoonoses and Public Health. (2023) 70, no. 2, 166–170, 10.1111/zph.13008. 36370131 PMC10099784

[19] Trombetta C. M. , Stufano A. , and Biagini V. , et al.Seroprevalence of Influenza D Virus in Cattle Workers: An Occupational Health Perspective, Journal of Medical Virology. (2026) 98, no. 2, 10.1002/jmv.70817, e70817. 41586448

[20] Lim E. H. , Lim S. I. , and Kim M. J. , et al.First Detection of Influenza D Virus Infection in Cattle and Pigs in the Republic of Korea, Microorganisms. (2023) 11, no. 7, 10.3390/microorganisms11071751, 1751. 37512923 PMC10386134

[21] Faccini S. , De Mattia A. , and Chiapponi C. , et al.Development and Evaluation of a New Real-Time RT-PCR Assay for Detection of Proposed Influenza D Virus, Journal of Virological Methods. (2017) 243, 31–34, 10.1016/j.jviromet.2017.01.019. 28153610 PMC7113724

[22] Hayakawa J. , Masuko T. , Takehana T. , and Suzuki T. , Genetic and Antigenic Characterization and Retrospective Surveillance of Bovine Influenza D Viruses Identified in Hokkaido, Japan From 2018 to 2020, Viruses. (2020) 12, no. 8, 10.3390/v12080877, 877. 32796617 PMC7472347

[23] Katoh K. and Standley D. M. , MAFFT Multiple Sequence Alignment Software Version 7: Improvements in Performance and Usability, Molecular Biology and Evolution. (2013) 30, no. 4, 772–780, 10.1093/molbev/mst010. 23329690 PMC3603318

[24] Katoh K. , Rozewicki J. , and Yamada K. D. , MAFFT Online Service: Multiple Sequence Alignment, Interactive Sequence Choice and Visualization, Briefings in Bioinformatics. (2019) 20, no. 4, 1160–1166, 10.1093/bib/bbx108. 28968734 PMC6781576

[25] Hall B. G. , Building Phylogenetic Trees from Molecular Data with MEGA, Molecular Biology and Evolution. (2013) 30, no. 5, 1229–1235, 10.1093/molbev/mst012. 23486614

[26] Martin D. P. , Murrell B. , Golden M. , Khoosal A. , and Muhire B. , RDP4: Detection and Analysis of Recombination Patterns in Virus Genomes, Virus Evolution. (2015) 1, no. 1, 10.1093/ve/vev003, vev003. 27774277 PMC5014473

[27] Rambaut A. , Lam T. T. , Max Carvalho L. , and Pybus O. G. , Exploring the Temporal Structure of Heterochronous Sequences Using TempEst (formerly Path-O-Gen), Virus Evolution. (2016) 2, no. 1, 10.1093/ve/vew007, vew007. 27774300 PMC4989882

[28] Drummond A. J. and Rambaut A. , BEAST: Bayesian Evolutionary Analysis by Sampling Trees, BMC Evolutionary Biology. (2007) 7, no. 1, 10.1186/1471-2148-7-214, 214. 17996036 PMC2247476

[29] Limaye S. , Lohar T. , and Dube H. , et al.Rapid Evolution Leads to Extensive Genetic Diversification of Cattle Flu Influenza D Virus, Communications Biology. (2024) 7, no. 1, 10.1038/s42003-024-06954-4, 1276. 39375524 PMC11458855

[30] Baele G. , Lemey P. , Bedford T. , Rambaut A. , Suchard M. A. , and Alekseyenko A. V. , Improving the Accuracy of Demographic and Molecular Clock Model Comparison While Accommodating Phylogenetic Uncertainty, Molecular Biology and Evolution. (2012) 29, no. 9, 2157–2167, 10.1093/molbev/mss084. 22403239 PMC3424409

[31] Lemey P. , Rambaut A. , Drummond A. J. , Suchard M. A. , and Fraser C. , Bayesian Phylogeography Finds Its Roots, PLoS Computational Biology. (2009) 5, no. 9, 10.1371/journal.pcbi.1000520, e1000520. 19779555 PMC2740835

[32] Pond S. L. K. , Frost S. D. W. , and Muse S. V. , HyPhy: Hypothesis Testing Using Phylogenies, Bioinformatics. (2005) 21, no. 5, 676–679, 10.1093/bioinformatics/bti079. 15509596

[33] Weaver S. , Shank S. D. , Spielman S. J. , Li M. , Muse S. V. , and Kosakovsky Pond S. L. , Datamonkey 2.0: A Modern Web Application for Characterizing Selective and Other Evolutionary Processes, Molecular Biology and Evolution. (2018) 35, no. 3, 773–777, 10.1093/molbev/msx335. 29301006 PMC5850112

[34] Murrell B. , Weaver S. , and Smith M. D. , et al.Gene-Wide Identification of Episodic Selection, Molecular Biology and Evolution. (2015) 32, no. 5, 1365–1371, 10.1093/molbev/msv035. 25701167 PMC4408417

[35] Smith M. D. , Wertheim J. O. , Weaver S. , Murrell B. , Scheffler K. , and Kosakovsky Pond S. L. , Less Is More: An Adaptive Branch-Site Random Effects Model for Efficient Detection of Episodic Diversifying Selection, Molecular Biology and Evolution. (2015) 32, no. 5, 1342–1353, 10.1093/molbev/msv022. 25697341 PMC4408413

[36] Murrell B. , Wertheim J. O. , Moola S. , Weighill T. , Scheffler K. , and Kosakovsky Pond S. L. , Detecting Individual Sites Subject to Episodic Diversifying Selection, PLoS Genetics. (2012) 8, no. 7, 10.1371/journal.pgen.1002764, e1002764. 22807683 PMC3395634

[37] Kosakovsky Pond S. L. and Frost S. D. W. , Not So Different After All: A Comparison of Methods for Detecting Amino Acid Sites Under Selection, Molecular Biology and Evolution. (2005) 22, no. 5, 1208–1222, 10.1093/molbev/msi105. 15703242

[38] Mekata H. , Yamamoto M. , and Hamabe S. , et al.Molecular Epidemiological Survey and Phylogenetic Analysis of Bovine Influenza D Virus in Japan, Transboundary and Emerging Diseases. (2018) 65, no. 2, e355–e360, 10.1111/tbed.12765. 29143492

[39] Ferguson L. , Eckard L. , and Epperson W. B. , et al.Influenza D Virus Infection in Mississippi Beef Cattle, Virology. (2015) 486, 28–34, 10.1016/j.virol.2015.08.030. 26386554 PMC4710178

[40] Horimoto T. , Hiono T. , and Mekata H. , et al.Nationwide Distribution of Bovine Influenza D Virus Infection in Japan, PLoS One. (2016) 11, no. 9, 10.1371/journal.pone.0163828, e0163828. 27682422 PMC5040247

[41] Kwaśnik M. , Rola J. , Larska M. , and Rożek W. , Serological Evidence of Influenza D Virus Circulation Among Cattle in Poland, Journal of Veterinary Research. (2025) 69, no. 3, 305–311, 10.2478/jvetres-2025-0048. 41064413 PMC12503218

[42] Alvarez I. , Banihashem F. , and Persson A. , et al.Detection and Phylogenetic Characterization of Influenza D in Swedish Cattle, Viruses. (2025) 17, no. 1, 10.3390/v17010017, 17. PMC11768518 39861806

[43] Chiapponi C. , Faccini S. , and Fusaro A. , et al.Detection of a New Genetic Cluster of Influenza D Virus in Italian Cattle, Viruses. (2019) 11, no. 12, 10.3390/v11121110, 1110. 31801277 PMC6949953

[44] Gorin S. , Richard G. , and Hervé S. , et al.Characterization of Influenza D Virus Reassortant Strain in Swine From Mixed Pig and Beef Farm, France, Emerging Infectious Diseases. (2024) 30, no. 8, 1672–1676, 10.3201/eid3008.240089. 39043445 PMC11286067

[45] Mazzetto E. , Bortolami A. , and Fusaro A. , et al.Replication of Influenza D Viruses of Bovine and Swine Origin in Ovine Respiratory Explants and Their Attachment to the Respiratory Tract of Bovine, Sheep, Goat, Horse, and Swine, Frontiers in Microbiology. (2020) 11, 10.3389/fmicb.2020.01136, 1136. 32523585 PMC7261881

