# Supplementary material for: Genetic and Phylogenetic Characterization of Influenza D Viruses From South Korean Cattle, 2022–2023
Source: Transbound Emerg Dis. 2026 Aug 5;2026:6780142. doi: 10.1155/tbed/6780142 (PMC13439223; doi:10.1155/tbed/6780142)
Supplement: Supplementary file 1 [file TBED-2026-6780142-s001.docx]

**Supplementary Table S1. Primers and probes used in this study**

| **Target** | **Primer / Probe** | **Sequence (5' → 3')** | **Position** | **Reference** |
| --- | --- | --- | --- | --- |
| **PB2** | F1 | GCATAAGCAGAGGATGTCACTAC | 2-24 | (1) |
|  | R1 | GCCTTACCCATTTTAGCAGCAC | 1350-1371 |  |
|  | F2 | GAAGGTCTGAAGGCCTAGATTG | 1171-1192 |  |
|  | R2 | GCAGTAGCAAGAGGATTTTTTCAATGTGC | 2335-2363 |  |
| **PB1** | F1 | GGCATAAGCAGAGGATTTTATAAC | 1-24 | (1) |
|  | R1 | CAGTTCCTTCTGGCCAATGCTTC | 1313-1335 |  |
|  | F2 | GAACCTTACATGGATGGAGAGTGC | 1205-1227 |  |
|  | R2 | AGCAGTAGCAAGAGGATTTTTCTG | 2307-2330 |  |
|  | F | TGGATGGAGAGTGCTGCTTC | 1190-1209 | (2) |
|  | R | GCCAATGCTTCCTCCCTGTA | 1279-1298 |  |
|  | Probe | FAM-CATGTTAAACATTCCCATCAGCATTCCT-BHQ1 | 1218-1245 |  |
| **P3** | F1 | GGCATAAGCAGGAGATTTAGAA | 1-22 | (1) |
|  | R1 | CATTAAACCATGTTGGGAACCCCC | 1149-1172 |  |
|  | F2 | GCCTCCAAGAAAATACAGGAGG | 1064-1085 |  |
|  | R2 | GCAGTAGCAAGGAGATTTTTAAC | 2172-2194 |  |
| **HEF** | F1 | GCATAAGCAGGAGATTTTCAAAG | 2-24 | (1) |
|  | R1 | CCATGGTTGTCATCTGCTTCTCCT | 1128-1151 |  |
|  | F2 | CTGAGTGGTCAGCTTCACGAAGAT | 1031-1054 |  |
|  | R2 | GCAGTAGCAAGGAGATTTTTTCTAAG | 2023-2048 |  |
| **NP** | F1 | GCATAAGCAGGAGATTATTAAGCA | 2-25 | (1) |
|  | R1 | CTGCATCTCCCACAATTCCCTT | 994-1015 |  |
|  | F2 | CTGGAGTAAGAGCCTTTATGGC | 932-953 |  |
|  | R2 | GCAGTAGCAAGGAGATTTTTTG | 1753-1774 |  |
| **M** | F | GCATAAGCAGAGGATATTTTTGACGC | 2-27 | (1) |
|  | R | GCAGTAGCAAGAGGATTTTTTCGCG | 1194-1218 |  |
| **NS** | F | GCATAAGCAGGGGTGTACAATTTC | 2-25 | (1) |
|  | R | GCAGTAGCAAGGGGTTTTTTCATA | 843-867 |  |
| **SP6** | F | TATTTAGGTGACACTATAG | - | (3) |
| **T7** | R | TAATACGACTCACTATAGGG | - | (3) |
| **M13** | F | TGTAAAACGACGGCCAGT | - | (4) |
|  | R | CAGGAAACAGCTATGAC | - |  |

**Supplementary Table S2. Reference influenza D virus sequences retrieved from the GenBank database.**
Supplementary Table S2 is provided as a separate Excel file.

**Supplementary Table S3. Molecular clock models and nucleotide substitution models used for Bayesian phylogenetic analyses of each IDV genomic segment**

| **Gene segment** | **Model** | |
| --- | --- | --- |
|  | **Clock Model** | **Nucleotide substitution model** |
| PB2 | UCLN | TN93+G |
| PB1 | UCLN | TN93+G |
| P3 | Strict | TN93+G |
| HEF | UCLN | HKY+G |
| NP | UCLN | TN93+G |
| M | UCLN | HKY+G |
| NS | Strict | HKY+G |
| UCLN, uncorrelated lognormal relaxed molecular clock; Strict, strict molecular clock; HKY, Hasegawa–Kishino–Yano model; TN93, Tamura–Nei 93 model; +G, gamma-distributed among-site rate heterogeneity | | |

**Supplementary Figure S1. Maximum-likelihood phylogenetic tree of the PB2 gene segment of influenza D viruses**

Maximum-likelihood phylogenetic tree of the PB2 gene segment of influenza D viruses. The six South Korean IDV strains are indicated by red circles. Branch colors represent the major IDV lineages: D/Oklahoma (green), D/660 (blue), D/CA2019 (cyan), D/Yama2016 (gray), and D/Yama2019 (red). Bootstrap values ≥80% are shown at nodes. The scale bar indicates nucleotide substitutions per site.

**Supplementary Figure S2. Maximum-likelihood phylogenetic tree of the PB1 gene segment of influenza D viruses**

Maximum-likelihood phylogenetic tree of the PB1 gene segment of influenza D viruses. The six South Korean IDV strains are indicated by red circles. Branch colors represent the major IDV lineages: D/Oklahoma (green), D/660 (blue), D/CA2019 (cyan), D/Yama2016 (gray), and D/Yama2019 (red). Bootstrap values ≥80% are shown at nodes. The scale bar indicates nucleotide substitutions per site.

**Supplementary Figure S3. Maximum-likelihood phylogenetic tree of the P3 gene segment of influenza D viruses**

Maximum-likelihood phylogenetic tree of the P3 gene segment of influenza D viruses. The six South Korean IDV strains are indicated by red circles. Branch colors represent the major IDV lineages: D/Oklahoma (green), D/660 (blue), D/CA2019 (cyan), D/Yama2016 (gray), and D/Yama2019 (red). Bootstrap values ≥80% are shown at nodes. The scale bar indicates nucleotide substitutions per site.

**Supplementary Figure S4. Maximum-likelihood phylogenetic tree of the NP gene segment of influenza D viruses**

Maximum-likelihood phylogenetic tree of the NP gene segment of influenza D viruses. The six South Korean IDV strains are indicated by red circles. Branch colors represent the major IDV lineages: D/Oklahoma (green), D/660 (blue), D/CA2019 (cyan), D/Yama2016 (gray), and D/Yama2019 (red). Bootstrap values ≥80% are shown at nodes. The scale bar indicates nucleotide substitutions per site.

**Supplementary Figure S5. Maximum-likelihood phylogenetic tree of the M gene segment of influenza D viruses**

Maximum-likelihood phylogenetic tree of the M gene segment of influenza D viruses. The six South Korean IDV strains are indicated by red circles. Branch colors represent the major IDV lineages: D/Oklahoma (green), D/660 (blue), D/CA2019 (cyan), D/Yama2016 (gray), and D/Yama2019 (red). Bootstrap values ≥80% are shown at nodes. The scale bar indicates nucleotide substitutions per site.

**Supplementary Figure S6. Maximum-likelihood phylogenetic tree of the NS gene segment of influenza D viruses**

Maximum-likelihood phylogenetic tree of the NS gene segment of influenza D viruses. The six South Korean IDV strains are indicated by red circles. Branch colors represent the major IDV lineages: D/Oklahoma (green), D/660 (blue), D/CA2019 (cyan), D/Yama2016 (gray), and D/Yama2019 (red). Bootstrap values ≥80% are shown at nodes. The scale bar indicates nucleotide substitutions per site.

**Supplementary Figure S7. Temporal signal assessment of IDV genomic segments by root-to-tip regression**

Genetic divergence from the root was plotted against sampling year for each segment: (A) PB2, (B) PB1, (C) P3, (D) HEF, (E) NP, (F) M, and (G) NS. Each dot represents an individual IDV sequence, and the fitted regression line indicates the association between sampling time and genetic divergence. The slope, correlation coefficient, and R² value are shown within each panel.

**Supplementary Figure S8. Bayesian maximum clade credibility tree of the PB2 gene segment of influenza D viruses**

The six South Korean IDV strains clustered within the D/Yama2019 lineage, shown in red. Major IDV lineages are distinguished by branch color: D/Oklahoma, green; D/660, blue; D/CA2019, cyan; D/Yama2016, gray; and D/Yama2019, red. Node labels indicate estimated divergence times, and horizontal bars represent 95% highest posterior density intervals. Node colors indicate posterior probabilities. The time axis is shown in calendar years.

**Supplementary Figure S9. Bayesian maximum clade credibility tree of the PB1 gene segment of influenza D viruses**

The six South Korean IDV strains clustered within the D/Yama2019 lineage, shown in red. Major IDV lineages are distinguished by branch color: D/Oklahoma, green; D/660, blue; D/CA2019, cyan; D/Yama2016, gray; and D/Yama2019, red. Node labels indicate estimated divergence times, and horizontal bars represent 95% highest posterior density intervals. Node colors indicate posterior probabilities. The time axis is shown in calendar years.

**Supplementary Figure S10. Bayesian maximum clade credibility tree of the P3 gene segment of influenza D viruses**

The six South Korean IDV strains clustered within the D/Yama2019 lineage, shown in red. Major IDV lineages are distinguished by branch color: D/Oklahoma, green; D/660, blue; D/CA2019, cyan; D/Yama2016, gray; and D/Yama2019, red. Node labels indicate estimated divergence times, and horizontal bars represent 95% highest posterior density intervals. Node colors indicate posterior probabilities. The time axis is shown in calendar years.

**Supplementary Figure S11. Bayesian maximum clade credibility tree of the NP gene segment of influenza D viruses**

The six South Korean IDV strains clustered within the D/Yama2019 lineage, shown in red. Major IDV lineages are distinguished by branch color: D/Oklahoma, green; D/660, blue; D/CA2019, cyan; D/Yama2016, gray; and D/Yama2019, red. Node labels indicate estimated divergence times, and horizontal bars represent 95% highest posterior density intervals. Node colors indicate posterior probabilities. The time axis is shown in calendar years.

**Supplementary Figure S12. Bayesian maximum clade credibility tree of the M gene segment of influenza D viruses**

The six South Korean IDV strains clustered within the D/Yama2019 lineage, shown in red. Major IDV lineages are distinguished by branch color: D/Oklahoma, green; D/660, blue; D/CA2019, cyan; D/Yama2016, gray; and D/Yama2019, red. Node labels indicate estimated divergence times, and horizontal bars represent 95% highest posterior density intervals. Node colors indicate posterior probabilities. The time axis is shown in calendar years.

**Supplementary Figure S13. Bayesian maximum clade credibility tree of the NS gene segment of influenza D viruses**

The six South Korean IDV strains clustered within the D/Yama2019 lineage, shown in red. Major IDV lineages are distinguished by branch color: D/Oklahoma, green; D/660, blue; D/CA2019, cyan; D/Yama2016, gray; and D/Yama2019, red. Node labels indicate estimated divergence times, and horizontal bars represent 95% highest posterior density intervals. Node colors indicate posterior probabilities. The time axis is shown in calendar years.

**References**

1.	Hayakawa J, Masuko T, Takehana T, Suzuki T. Genetic and Antigenic Characterization and Retrospective Surveillance of Bovine Influenza D Viruses Identified in Hokkaido, Japan from 2018 to 2020. Viruses. 2020;12. 10.3390/v12080877

2.	Faccini S, De Mattia A, Chiapponi C, Barbieri I, Boniotti MB, Rosignoli C, et al. Development and evaluation of a new Real-Time RT-PCR assay for detection of proposed influenza D virus. J Virol Methods. 2017;243:31–4. 10.1016/j.jviromet.2017.01.019

3.	Schenborn ET, Mierendorf RC, Jr. A novel transcription property of SP6 and T7 RNA polymerases: dependence on template structure. Nucleic Acids Res. 1985;13:6223–36. 10.1093/nar/13.17.6223

4.	Yanisch-Perron C, Vieira J, Messing J. Improved M13 phage cloning vectors and host strains: nucleotide sequences of the M13mp18 and pUC19 vectors. Gene. 1985;33:103–19. 10.1016/0378-1119(85)90120-9
